# The Hospital World

**Published:** 1910-06-11

**Authors:** 


### The Hospital World.

### Personalia.

Sir Richard Douglas Powell has resigned the chairmanship and the membership of the consulting staff of the King Edward VII. Sanatorium.

Dr. Theodore Williams has become chairman of the consulting staff of the King Edward VII. Sanatorium, and Dr. Bertram Dawson has been appointed hon. secretary.

The Aberdeen Royal Infirmary, which lost a skilled and faithful member of its medical staff by the death of Dr. Dalgarno, has sent to Mrs. Dalgarno an expression of the loss and sympathy it feels. For more than fourteen years Dr. Dalgarno had acted on the staff of the Infirmary, his position of chief anæsthetist being a most responsible one. During this long period Dr. Dalgarno won respect for his skill and affection for his personality, and the regret which every Aberdeen man feels in his death is both a personal and a professional one. The post of chief anæsthetist is nowadays peculiarly responsible, and to have carried its responsibilities for fourteen years with such honourable success as Dr. Dalgarno did is a proof of conscientiousness and ability that Aberdeen will be grateful for.

By the death of Mr. Alfred Colson, the manager of the Leicester Corporation Gas and Electric Light Departments, the Leicester Infirmary loses a member of the board whose technical knowledge and extensive practical experience have been of great value to the institution, especially in connection with the administrative details. Not only was Mr. Colson of great service as a member of the boards and Committees, but he was a powerful advocate of the cause of the institution. It was largely due to his influence, and it was under his generalship, that the "Murdock Centenary Cot" was endowed in the Children's Hospital by the employés of the Gas and Electric Lighting Departments, and systematic weekly contributions made amongst the officials, staff, and workpeople of his departments for the Infirmary and Children's Hospital. For the latter Mr. Colson regarded it a privilege to be able to send an annual cheque, usually for £250. The borough of Leicester loses an invaluable officer, and the Leicester Infirmary a staunch friend by Mr. Colson's death at the age of 61.

For sixteen years the Rev. F. N. Harvey has been chaplain to the Royal South Hants and Southampton Hospital. It was felt that his work could not go unacknowledged, and also that Mr. Harvey would value some memento of his life there, which he could carry away with him to his new benefice of Whitchurch. So steps were taken, with the result that Mr. Harvey was presented with a cheque for £50, and a silver plate fastened to a revolving bookcase, which was inscribed as follows: "Presented to the Rev. F. N. Harvey, M.A., by the governors and hon. medical staff of the R.S.H. and Southampton Hospital, as a token of esteem after sixteen years' work as chaplain. May 1910." In acknowledging the presentation, Mr. Harvey said: "I have tried during the time of my chaplaincy here to keep in mind the fact that the hospital is the place where, primarily, the body is to be cared for—the chaplain's work is a complementary one." The words of other speakers, however, including those of Mr. C. T. Wilson, who presided, showed that Mr. Harvey had made his work complementary chiefly in the sense that it was a course of personal service the hospital patients regarded as their happiness to receive.

Mr. W. Sutton, Jun., Secretary of the Building Committee of the Royal Victoria Infirmary, Newcastle, has been presented with a tea and coffee set and salver for his continued services on that committee. In making the presentation Sir Riley Lord said: "The Queen's Commemoration Fund was founded in 1896, when I was Mayor of the city, and Mr. Sutton became the Secretary, and subsequently Mr. Sutton was appointed Secretary of the Building Committee. The work which was done extended over a period of close on fourteen years, during which time I was in very close touch with Mr. Sutton, who made a most excellent Secretary. Mr. Sutton displayed considerable ability and always got on well with the whole of the members of the committee, who now desired to show their appreciation of his services." To be for fourteen years on a building committee is a triumph in itself. No institutional work, perhaps, is more difficult or better deserves acknowledgment than this.

### Elections and Appointments.

Mr. J. D. Hodges has been chosen to represent Heston and Isleworth on the Richmond Joint Hospital Committee.

Mr. W. Dalglish-Bellasis has been re-elected President of the Ashby-de-la-Zouch and District Cottage Hospital.

Mr. Thomas Buckham and Mr. George Young have been elected life governors at the recent quarterly court of the Royal Victoria Infirmary, Newcastle.

Mr. James A. C. Brumwell has been re-elected chairman, and Mr. W. R. Jameson has been reappointed vice-chairman of the Gosforth, Newburn, and Castle Ward Joint Hospital Authority.

Mr. Cuthbert Blundell, of Halsall, has been elected president of the Ormskirk Dispensary and Cottage Hospital in succession to the late Earl of Lathom. In this he makes the post, as it were, a hereditary one, for the late Canon Blundell, his father, had held the office in his life. We have often pointed out how much institutions and families gain from such extended connections as these. Mr. R. E. Hattersley (hon. secretary) and Mr. Baxendale (hon. treasurer) have been re-elected also.

The Leamington Hospital Saturday Committee includes, by the recent election, Mrs. Canning, Mrs. Joyce, Mrs. Middleton, Mrs. Spracklin, Mr. Walker, Miss Wimbush, Mr. J. W. Barnes, Mr. T. W. Bartlett, Mr. A. R. Baylise, Mr. H. Berry, Mr. C. Canning, Mr. H. H. Ellis, Mr. J. Haycock, Mr. W. Jackman, Mr. C. Middleton, Mr. J. Nutting, Mr. F. Robinson, Mr. N. G. Robinson, Mr. G. Rooke, Mr. J. Spracklin, Mr. W. G. Spracklin, Mr. W. M. Shuff, Mr. G. C. Ward, Mr. T. Wimbush, Mr. H. L. Chapman, Mr. C. Reeves, Mr. G. Paine, and Mr. W. T. Chattaway.

Mrs. Love, of Hawkhills, now feels unable to continue to maintain, at her own expense (as she has finely done for many years), the St. Monica Hospital at Easingwold. But she is willing to give the building and the hospital equipment free of charge, should the people of Easingwold and district carry on the institution by public subscription. So willing has the neighbourhood proved, having subscribed £210 already, that a committee has been appointed to make arrangements for the reopening of the hospital as a public institution, consisting of the chairman, Alderman Hawking, Dr. Hicks, Dr. Mills, Mr. G. Knowles, Mr. J. C. Bannister, Mr. T. Cowling, Mr. R. Souter, Major Miles J. Stapylton, and Dr. Elvins, to draw up rules and regulations.

